# Enhanced C–H bond activation by tuning the local environment of surface lattice oxygen of MoO_3_†

**DOI:** 10.1039/d2sc01658c

**Published:** 2022-05-17

**Authors:** Chenggong Jiang, Xin Chang, Xianhui Wang, Zhi-Jian Zhao, Jinlong Gong

**Affiliations:** Key Laboratory for Green Chemical Technology of Ministry of Education, School of Chemical Engineering and Technology, Tianjin University Tianjin 300072 China zjzhao@tju.edu.cn jlgong@tju.edu.cn; Collaborative Innovation Center of Chemical Science and Engineering Tianjin 300072 China; Joint School of National University of Singapore and Tianjin University, International Campus of Tianjin University Binhai New City Fuzhou 350207 China; Haihe Laboratory of Sustainable Chemical Transformations Tianjin 300192 China

## Abstract

The lattice oxygen on transition metal oxides serves as a critical active site in the dehydrogenation of alkanes, whose activity is determined by electronic properties and environmental structures. Hydrogen affinity has been used as a universal descriptor to predict C–H bond activation, while the understanding of the environmental structure is ambiguous due to its complexity. This paper describes a combined theoretical and experimental study to reveal the activity of lattice oxygen species with different local structures, taking Mo-based oxides and C–H bond activation of low-carbon alkanes as model catalytic systems. Our theoretical work suggests that oxygen species with convex curvature are more active than those with concave curvature. Theoretically, we propose an interpretative descriptor, the activation deformation energy, to quantify the surface reconstruction induced by adsorbates with various environmental structures. Experimentally, a Mo-based polyoxometalate with the convex curvature structure shows nearly five times the initial activity than single-crystal molybdenum oxide with the concave one. This work provides theoretical guidance for designing metal oxide catalysts with high activity.

## Abstract 2

Tuning of the environmental structure near the lattice oxygen of molybdenum oxides can form a favorable spatial structure to enhance the intrinsic activity for C–H bond activation.

## Graphical abstract

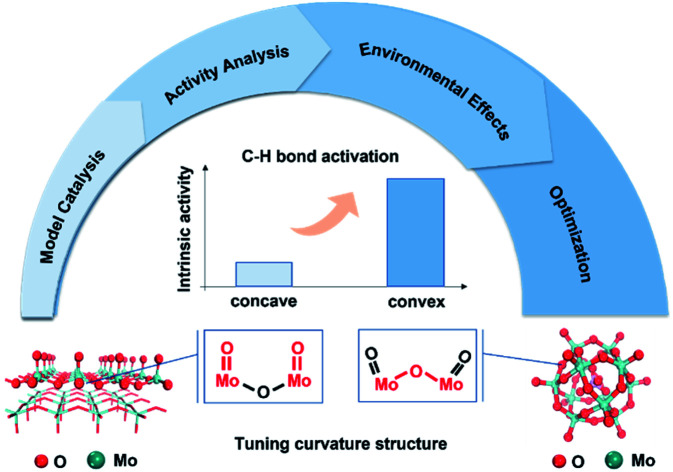

## Introduction

Developing highly effective alkane dehydrogenation catalysts is a critical technical challenge for producing olefins from shale gas, a rich source of light hydrocarbons (CH_4_, C_2_H_6_, and C_3_H_8_).^1^ The oxidation of alkanes begins with C–H bond activation, which is generally considered the rate-determining step for the whole reaction.^2^ Due to the inert C–H bond of low-carbon alkanes, effective and selective activation of the C–H bond of low-carbon alkanes has always been challenging in designing such catalysts.^1^ Despite non-oxidative dehydrogenation being the industrial technology, the process is limited to using costly platinum or toxic chromium catalysts. Among the non-noble catalysts explored, some reducible transition metal oxides are promising ones.^3–5^ The surface lattice oxygen is believed to be a key active site for alkane dehydrogenation. The activation of alkane molecules occurs through the Mars–van Krevelen mechanism, which entails oxidation of hydrocarbons with reduction of the oxide surface through losing surface lattice oxygen species.^6^ Generally, the activity of lattice oxygen is determined by the electronic structure and the surrounding environment. However, because a single surface lattice oxygen can activate the C–H bond, many descriptors are proposed to predict the activity of oxygen species solely, such as hydrogen affinity (*E*_H_),^7^ oxygen vacancy formation energy,^8^ and O-2p band center.^9^ In contrast, recent studies clearly showed the importance of the local environment near the active site, especially for metal–organic framework (MOF-based) materials^10^ and zeolite catalysts.^11^ The catalytic microenvironment could form a unique spatial structure accompanied by various electronic properties around the active catalytic sites, which plays an essential role in regulating the catalytic performance.^12,13^ However, there is a lack of clear and general comprehension of how the local environment affects the activity. Therefore, an insightful understanding of how the local environment around the lattice oxygen is critical for designing transition metal oxide catalysts with effective C–H bond activation is essential.

Molybdenum trioxide (MoO_3_) is a reducible oxide used in crucial partial oxidation reactions, such as methanol production^14^,^15^ alkene metathesis,^16^ and hydrocracking.^16^ In particular, it has been used in many studies of C–H bond activation of alkanes and alcohols. For example, Choksi *et al.* studied the partial oxidation of methanol on MoO_3_ by DFT and microkinetic simulation and elaborated the universal single crystal model reaction for catalytic kinetic modeling.^17^ Amakawa *et al.* used molybdenum oxides as a model system to study the strain effect on the activation of propane oxidation.^18^ Yao *et al.* investigated the kinetics of oxidative and non-oxidative dehydrogenation of ethane on molybdenum oxide and developed a generalized mechanistic framework.^19^ These studies have shown that molybdenum oxide is a common and suitable model system for studying C–H bond activation.

**Fig. 1:**
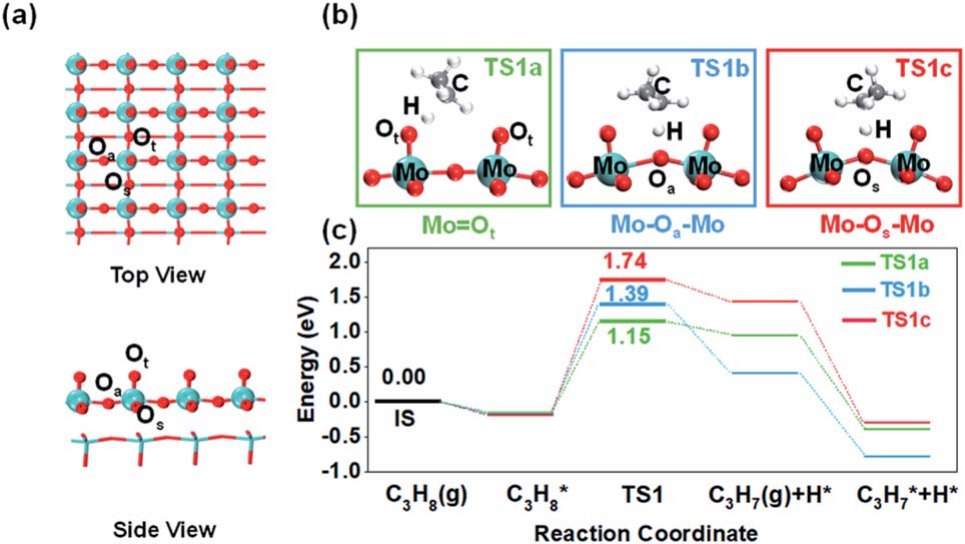
Identification of different oxygen species activity and propane dehydrogenation mechanism. (a) Optimized structures of the α-MoO_3_ (010) pristine surface and three distinctive lattice oxygen species. (b) C–H bond activation transition states on three lattice oxygen species. (c) Potential energy diagram and transition state structures of the first step, C–H bond activation, over three oxygen species.

This paper describes the influence of the environmental structure of different oxygen species on the reactivity of propane dehydrogenation employing α-MoO_3_ as a model of transition metal oxide catalysts. α-MoO_3_ with an orthorhombic cell and *pbmn* space group is the most common crystal structure in molybdenum oxides. Previous studies have shown that the (010) crystal plane is the most stable and dominant.^20^ The α-MoO_3_ (010) crystal plane provides three distinct oxygen atoms: terminal (O_t_), asymmetrical bridging (O_a_), and symmetrical bridging (O_s_) oxygen atoms (Fig. 1a), establishing a great platform with various environmental structures to study the effect. However, molybdenum oxide's low C–H bond activation ability restricts its industrial application. Our theoretical study shows that the bridging oxygen with higher C–H breaking ability is limited by the unfavorable steric structure, which is the main reason for its poor surface activity. Furthermore, through theoretical calculations and experiments, we demonstrated that optimizing such a spatial structure near the surface lattice oxygen can greatly improve its C–H bond activation ability by using a molybdenum-based Keggin-polyoxometalate (POM), one kind of cluster-type molybdenum oxide, as the model catalyst.

## Results and discussion

### Surface lattice oxygen activity on the α-MoO_3_ model

Firstly, it is imperative to investigate the activity differences between different oxygen species. Many previous microkinetic and experimental studies on propane dehydrogenation have demonstrated that the first step of C–H bond activation on the methylene of propane is the rate-determining step (RDS) of the overall dehydrogenation reaction.^21–24^ Therefore, methylene C–H activation is considered the most crucial elementary reaction. We calculated propane dehydrogenation's activation energies (*E*_a_) at three different oxygen sites (Fig. 1). The terminal oxygen (Mo=O_t_) was calculated to be the most active site (*E*_a_ = 1.15 eV), while asymmetric bridging oxygen (Mo–O_a_–Mo) (*E*_a_ = 1.39 eV) was less active. The dehydrogenation of symmetric bridging oxygen (Mo–O_s_–Mo) was hindered by too high an energy barrier (*E*_a_ = 1.74 eV). Consistently, the oxygen vacancy formation energies of O_t_ and O_a_ (Table S2†) are similar, while the oxygen vacancy formation energy of O_s_ is much higher. The above results indicate that O_s_ is the most stable and inactive oxygen species. Therefore, Mo–O_s_–Mo will not be discussed in the following text unless stated. It is noteworthy that through differential charge density analysis, the metal–oxygen species participate in hydrogen abstraction as Lewis acid–base pairs (Fig. S4†), which means that the lattice oxygen (O^2−^) acts as a Lewis base site for accepting protons and eventually the metal cations as Lewis acid sites accept electrons to be reduced during the activation of the C–H bond.

**Fig. 2:**
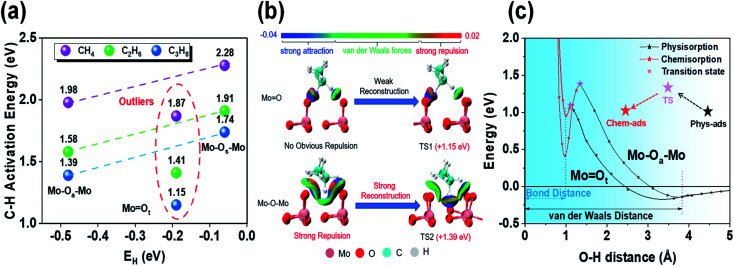
Effects of different local environmental geometries on oxygen species activity. (a) Comparison of the first step C–H bond activation energy and *E*_H_ of C_1_–C_3_ alkanes on different oxygen species. (b) Non-covalent interaction analysis for PDH at Mo=O_t_ and Mo–O_a_–Mo *via* the independent gradient model (IGM). In the transition state distance: unreconstructed surface and inactivated C_3_H_8_ (left); adsorbate-induced reconstructed surface and activated C_3_H_8_ (right). (c) Analysis of the first step, dehydrogenation of propane, in Mo=O_t_ and Mo–O_a_–Mo *via* the crossing potential model.

For a simpler prediction of oxygen activity, Nørskov and co-workers proposed that hydrogen affinity (*E*_H_) is one universal descriptor of reactivity for radical-like hydrocarbon activation.^7^ They found that *E*_H_ is proportional to the C–H activation energy of alkanes in various materials. We also calculated *E*_H_ at three oxygen sites and tried to correlate them with propane activation energy. We found that the *E*_H_ sequence of the three oxygen species was: Mo–O_a_–Mo (*E*_H_ = −0.48 eV) < Mo=O_t_ (*E*_H_ = −0.19 eV) < Mo–O_s_–Mo (*E*_H_ = −0.06 eV). However, when comparing the *E*_H_ and C–H activation energies of Mo–O_a_–Mo and Mo=O_t_, there was an anomaly (C–H activation energy: Mo=O_t_ < Mo–O_a_–Mo). To search for potential and common patterns, we made the same calculation and comparison for methane and ethane (the homologs of propane) at three active sites (Fig. 2a). Anomalies exist in all of them. Such an anomaly indicates that other factors besides the lattice oxygen greatly affect the activation of propane when compared with terminal oxygen and bridging oxygen. We speculated that this is due to the environmental effect around different oxygen species.

### Environmental effects on the activity of lattice oxygen species

To support our argument, we used the independent gradient model (IGM) to visually analyze the non-covalent interactions between molecular fragments and active sites to qualitatively show the different environmental effects before and after propane molecules' activation by bridging oxygen (Mo–O_a_–Mo) and terminal oxygen (Mo=O_t_) (Fig. 2b). The results showed that when propane was close to Mo=O_t_, strong attraction (blue) and weak van der Waals force (green) were dominant, while strong repulsion (red) appeared at Mo–O_a_–Mo. Furthermore, when comparing the transition states, Mo=O_t_ underwent no evident reconstruction. However, there was obvious reconstruction in Mo–O_a_–Mo, and the previous strong repulsion disappears completely in the reconstructed transition state structure. Strong attraction indicated that the C–H bond was about to break, and meanwhile the O–H bond was about to form, which, together with van der Waals force, constitutes stabilization.

Moreover, we used the crossing potential model to deeply understand how the environmental structure affected the C–H activation energy (Fig. 2c). The model showed that the potential energy varies with the distance from the adsorbate to the surface. It is generally believed that the activation of reactants would undergo a transition from physisorption to chemisorption.^25^ The crossing point of the two potential functions is exactly the transition state. The results showed that the C–H bond activation on the two active sites had almost the same O–H bond distance (∼0.98 Å). However, the energy of O_a_H was lower than that of O_t_H, which was the direct embodiment of the stronger hydrogen affinity of O_a_ than that of O_t_. Mo–O_a_–Mo is more active than Mo=O_t_ intrinsically. As the hydrogen affinity becomes exothermic, the chemisorption potential moves down as a whole, and meanwhile, the crossing point (transition state) also moves down. Therefore, there is a linear relationship between adsorption and activation energy (*i.e.*, Brønsted–Evans–Polanyi relationship).^26^

However, from the stable physisorption to the transition state, propane continues to approach the surface, causing some overlap of the electron clouds on the surface of propane and the catalyst. It will lead to repulsive interaction (*i.e.*, Born repulsion), which induces surface reconstruction by increasing the potential energy. Comparing the energy changes of both active sites, we found that the energy rise of Mo–O_a_–Mo is more obvious than that of Mo=O_t_. Combined with the results of IGM analysis, we can know that this stage is mainly due to the strong surface reconstruction in the process of C–H bond activation at the bridging oxygen (Mo–O_a_–Mo).

### Optimizing the oxygen activity by tuning the environmental structure

**Fig. 3:**
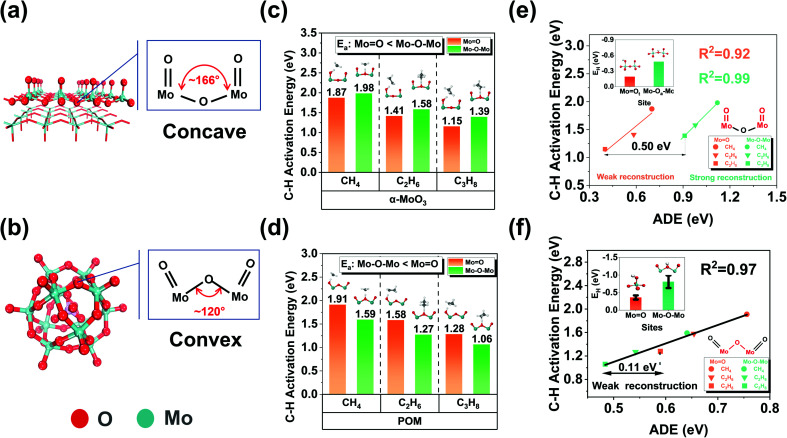
Design of more active catalysts by optimizing the microenvironment around the oxygen species. (a and b) Optimized structures of α-MoO_3_ (010) pristine surface and POM (H_3_PMo_12_O_40_) with concave and convex curvature structure characteristics, respectively. H atoms are hidden; (c and d) activation energy of CH_4_, C_2_H_6_, and C_3_H_8_ at Mo=O and Mo–O–Mo active sites with concave and convex curvature environmental structures. (e and f) Correlation between activation deformation energy and C–H activation energy of alkanes at the active sites of oxygen species with different structures. The red labels indicate the key active sites.

Such strong surface reconstruction revealed that the unfavorable environmental structure near α-MoO_3_ (010) Mo–O_a_–Mo originates from the concave local environment of Mo–O–Mo (Fig. 3a). Therefore, it is likely to optimize the steric spatial impact and enhance the catalytic activity to a certain extent. We noted that the molybdenum-based Keggin-polyoxometalate (POM) is an ideal model catalyst to verify our idea. Mo-based POMs (H_*n*_XMo_12_O_40_, X is the central heteroatom, such as P, Si, and Al) are kinds of metal oxide clusters with well-defined chemical composition and structure. POM has excellent thermal stability and redox properties, and there are many remarkable experimental and theoretical studies on the oxidative dehydrogenation of methanol^42,43^ and low-carbon alkanes.^44^ More importantly, POM has an optimized local environment (convex) of surface lattice oxygen compared to MoO_3_ and other comparable structural features (Fig. 3b and Table S3†). Phosphomolybdic acid hydrate (H_3_PMo_12_O_40_) is the most common Mo-based POM. Therefore, it is very suitable as a model comparison catalyst. Similar to α-MoO_3_, there are two kinds of oxygen species (Mo=O and Mo–O–Mo) on POM. We calculated the *E*_H_ of different oxygen species according to their symmetries (Fig. S5†). The results suggested that all the bridging oxygens (Mo–O–Mo) are stronger than terminal oxygens (Mo=O) in hydrogen affinity like α-MoO_3_. We selected the terminal oxygen and bridging oxygen closest to the average *E*_H_ as the representative of the average reactivity for the following investigation. Similarly, we compared the C–H activation energy for the first step dehydrogenation of methane, ethane, and propane on different oxygen species on α-MoO_3_ (Fig. 3c) and H_3_PMo_12_O_40_ (Fig. 3d). The results showed that in contrast to α-MoO_3_, all the C–H activation energies of Mo–O–Mo are lower than those of Mo=O. The results showed that the C–H bond activation activity of the bridging oxygen with convex curvature was higher than that of the bridging oxygen with concave curvature.

Moreover, to quantitatively describe the surface reconstruction induced by adsorbates mentioned above, we propose a new descriptor, the activation deformation energy (ADE), defined using the following equation:ADE = *E*(catalyst)_TS_ − *E*(catalyst)_IS_where TS and IS are short for the transition state and initial state, respectively, ADE is single-point energy with clear physical meaning, the energy increase caused by the surface reconstruction induced by the activated adsorbates. We correlated the C–H activation energies of the terminal oxygen and bridging oxygen on the surface of α-MoO_3_ and H_3_PMo_12_O_40_ with ADE (Fig. 3e and f) and found that there was a good linear relationship.

For α-MoO_3_, ADE (0.9–1.2 eV) on the Mo–O–Mo active site is significantly larger than that on Mo=O (0.4–0.7 eV), indicating that there is a strong reconstruction on the bridging oxygen, but not on the terminal oxygen; For POM, the surface reconstruction is relatively weak (0.48–0.75 eV) for both terminal oxygen and bridging oxygen. It is worth noting that the difference between ADEs of the same reactant at different active sites is almost the same. For example, it is 0.50 eV for α-MoO_3_ and 0.11 eV for H_3_PMo_12_O_40_. When comparing *E*_H_ and the constant difference, we found that for α-MoO_3_, *E*_H_ could not cover the difference, but for H_3_PMo_12_O_40_, it could. The surface reconstruction induced by H atom adsorption is almost negligible due to its tiny volume. However, the real reactants may induce strong surface reconstruction in the transition state, and such information is not included in the *E*_H_. Therefore, for some potential active sites (*e.g.*, Mo–O–Mo on α-MoO_3_), the obvious surface reconstruction induced by adsorbates is the fundamental reason why *E*_H_ cannot give an accurate prediction.

### Experimental verification

A thermogravimetric test (Fig. S6†) was done on the H_3_PMo_12_O_40_/Al_2_O_3_ catalyst to ensure consistency between the theoretical model and experimental structure. Then 723 K was determined to be the appropriate reaction temperature.

To investigate the structural stability of the theoretical model at the reaction temperature and the changes in the microenvironmental features of Mo–O–Mo, we did ab initio molecular dynamics simulation (AIMD) for 30 000 fs at 723 K for H_3_PMo_12_O_40_ (Fig. S7†). The results showed that the overall structure and energy are stable. Besides, the statistical average bond angle about the active site (Mo–O–Mo) is 127.5°, and its key convex curvature structural features remained unchanged.

**Fig. 4:**
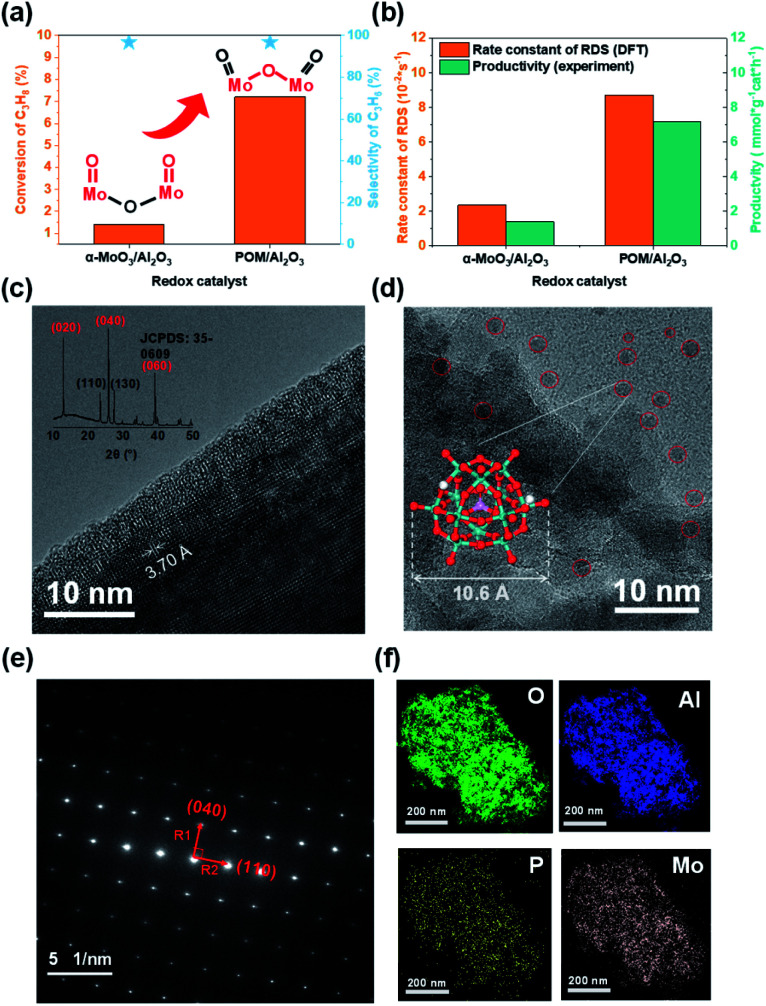
Experimental verification. (a) The instantaneous performance at the 5^th^ min of 20 wt% α-MoO_3_ physically dispersed on the Al_2_O_3_ support and 20 wt% POM(H_3_PMo_12_O_40_)/Al_2_O_3_. Reaction conditions: *T* = 723 K, 1.4 atm (C_3_H_8_ : N_2_ = 0.125) and 0.5 g sample. (b) DFT calculated rate constant of RDS at active sites (Mo=O for MoO_3_; Mo–O–Mo for H_3_PMo_12_O_40_) and experimentally measured productivity. (c) TEM and XRD for α-MoO_3_ (d) TEM for 20 wt% H_3_PMo_12_O_40_/Al_2_O_3_. (e) SAED for α-MoO_3_ sample. (f) EDS elemental mapping for 20 wt% H_3_PMo_12_O_40_/Al_2_O_3_.

To verify the theoretical results, we tested the initial performance of two Mo-based oxide model catalysts for propane dehydrogenation at 723 K (Fig. 4a). The propane conversion on H_3_PMo_12_O_40_/Al_2_O_3_ was nearly five times higher than on α-MoO_3_/Al_2_O_3_ without significantly decreasing propylene selectivity. The carbon balance was more than 98%, indicating that there was little coking. The results showed that the yield of propane dehydrogenation catalyzed by supported H_3_PMo_12_O_40_ was significantly better than that of α-MoO_3_ under the same test conditions.

Furthermore, to better explain the difference in the intrinsic activity of the active sites in the catalyst experiments, we calculated the activation free energy as well as the rate constants of the rate-determining step (RDS) for the active sites on the two model catalysts (Tables 1 and S4†). We found that the ratio of the rate constants of the RDS at the active sites of the two model catalysts and the experimentally observed productivity ratios are consistent in the order of magnitude (Fig. 4b).

**Table 1:** Comparison of Gibbs's activation free energy (Δ*G*^≠^) and rate constant (*k*) at different active sites

Catalyst	Site	Δ*G*^≠^^a^/eV	*k*(RDS)^b^/(s^−1^)
α-MoO_3_	Mo=O	2.08	2.35 × 10^−2^
H_3_PMo_12_O_40_	Mo–O–Mo	2.00	8.72 × 10^−2^

a Temperature: 723 K.

b RDS: C_3_H_8_(g) → C_3_H_7_(g) + H*.

TEM and XRD (X-ray diffraction) (Fig. 4c), and SAED (selected area electron diffraction) (Fig. 4e) confirmed the rationality of our theoretical model, that is, α-MoO_3_ was a lamellar structure and the (010) facet was the dominating plane. TEM (Fig. 4d) and energy-dispersive X-ray spectroscopy (EDS, Fig. 4f) for 20 wt% H_3_PMo_12_O_40_/Al_2_O_3_ showed that POM was uniformly dispersed on the alumina support.

## Conclusions

Our work showed that the C–H bond activation of alkanes could be significantly enhanced by tuning the spatial structure around the surface lattice oxygen of molybdenum oxides. Firstly, we explored the differences in the activity of different surface lattice oxygens and their nature using α-MoO_3_ (010). The activity of terminal oxygen (Mo=O) is higher than that of bridging oxygen (Mo–O–Mo) on the α-MoO_3_ (010) plane. However, the hydrogen affinity (*E*_H_) showed that Mo–O–Mo is the potential active site with higher intrinsic activity but limited by its unfavorable environmental structure with concave curvature.

Furthermore, we designed a cluster-type molybdenum oxide (*i.e.*, Keggin-type polyoxometalate, H_3_PMo_12_O_40_) with well-defined structures with concave curvature for Mo–O–Mo. We proved that such Mo–O–Mo would significantly enhance the C–H bond activation of low-carbon alkanes compared to α-MoO_3_ (010) by density functional theory calculations. Moreover, we found that a universal descriptor of hydrogen affinity (*E*_H_) cannot predict the real activity of the oxygen species with concave curvature. Therefore, a quantitative descriptor, activation deformation energy (ADE), is proposed to describe the surface reconstruction induced by adsorbates. ADE provides us with a quantitative method with clear physical meaning to help deeply understand how the influence of adsorbate-induced surface reconstruction affects the activation energy. Also, we found that *E*_H_ can give the correct qualitative activity prediction about lattice oxygen only when *E*_H_ is greater than ADE; otherwise, it cannot.

Finally, experimentally, we used alumina-supported H_3_PMo_12_O_40_ and α-MoO_3_ as contrasting catalysts to test the propane dehydrogenation performance and confirmed the obvious improvement (∼5 times) of propane activity and propylene productivity when using H_3_PMo_12_O_40_/Al_2_O_3_.

This work provided more insights into the influence of the environmental structure around lattice oxygen species on metal oxides and some guidance for designing highly active metal oxide catalysts by tuning the surface environment around lattice oxygen.

## Methods

The calculations were performed using the Vienna ab initio simulation program^27,28^ (VASP), version 5.4.4 using the projector augmented wave (PAW) method.^29–31^ Self-consistently calculated total energies were evaluated with the Perdew–Burke–Ernzerhof (PBE)^32^ functional. The Kohn–Sham equations were solved using a plane-wave basis set with a cutoff energy of 400 eV. The Gaussian smearing method was adopted with a width of 0.05 eV to determine how each wave function's partial occupancies are set. Dipole correction was considered where necessary. All calculations were spin-polarized unless otherwise stated.

For systems such as transition metal oxides, the electronic bandgap may be underestimated, and even a qualitatively wrong metallic ground state may be predicted. The DFT + *U* approach^33,34^ has been proved to study a large variety of strongly correlated compounds with considerable improvement concerning LDA or GGA results. The Hubbard *U* value was applied to the molybdenum centers in this work. By fitting the enthalpies of formation and reduction of MoO_3_, we determined that the appropriate *U*_eff_ (where *U*_eff_ = *U* − *J*) value is 2.0 eV, which is consistent with a previous report.^20^ The experimental data of enthalpy of formation and enthalpy of reduction were obtained from the NIST-JANAF database (Fig. S2†).

For bulk optimization, the sampling of the Brillouin zone was performed using a Monkhorst–Pack scheme^35^ of (4 × 1 × 4). The convergence of the *k*-point is confirmed with the H adsorption energy. All atoms in the one-bilayered slabs for 2 × 2 to 5 × 5 supercells were relaxed. Moreover, as a descriptor, oxygen vacancy formation energy was used to test the convergence and confirm that the appropriate model in the *XY* direction is 4 × 4.

For the *Z* direction, adsorption energies of some important intermediates on one-bilayered 2 × 2 and two-bilayered 2 × 2 slabs were nearly identical, with differences of less than 0.05 eV. Thus the one-bilayered slab was used to simulate the *Z* direction. Slabs were separated from their periodic images with 15 Å of vacuum (Fig. S3†).

All calculations are performed on the Mo-based Keggin POM (H_3_PMo_12_O_40_) clusters (diameter ∼1.1 nm) placed in the center of 2 × 2 × 2 nm^3^ cells to prevent the electron density overlap between clusters in adjacent cells. Gamma point (1 × 1 × 1) mesh was used to sample the first Brillouin zone. Dipole/quadrupole corrections were used for H_3_PMo_12_O_40_ to eliminate long-range electrostatic interactions and thus allow simulation of isolated clusters within periodic boundaries of VASP. The structures of reactants, products and stable intermediates were optimized until the atomic force was less than 0.05 eV Å^−1^. The other calculation details are consistent with MoO_3_.

Transition state geometries were located using the climbing image nudged elastic band method (CI-NEB)^36,37^ with total 6 images, followed by refinement with the dimer method.^38^ Vibrational frequencies, computed from the Hessian matrix under the harmonic approximation, were used to calculate zero-point energy (ZPE) corrections. Calculating the frequency of all transition state structures confirmed that they have only one imaginary frequency.

Independent gradient model (IGM) is a new electron density (ED)-based methodology developed by Lefebvre *et al.* to identify different types and strength of interactions between chemical fragments, especially weak non-covalent interactions (NCI), such as vdW interaction and hydrogen bonds.^39^ The expression of the ED gradient in terms of atomic components furnishes the basis for the Independent Gradient Model (IGM). Using an intra/inter uncoupling scheme, a descriptor (*δg*^inter^) is then derived that uniquely defines intermolecular interaction regions. For a detailed definition and discussion, please refer to the original ref. 39 All IGM analyses were carried out using Multiwfn software (version 3.8).^40^ The visualization of the 3D isosurface (isovalue = 0.01 a.u.) is realized by VMD software.^41^

## Data availability

The data that supports the findings of this study is available from the corresponding author upon reasonable request.

## Author contributions

J. G. conceived and coordinated the research. C. J. conducted the density functional theory calculations and wrote the draft. X. W. contributed to catalyst synthesis, catalytic experiments and characterization. C. J, X. C, and Z. Z. analysed the data. C. J., X. C., Z. Z. and J. G. wrote the manuscript. All authors discussed, commented on and revised the manuscript.

## Conflicts of interest

There are no conflicts to declare.

## Supplementary Material

SC-013-D2SC01658C-s001
