# Supplementary material for: Enhanced C–H bond activation by tuning the local environment of surface lattice oxygen of MoO3
Source: Chem Sci. 2022 May 17;13(25):7468–74. doi: 10.1039/d2sc01658c (PMC9241962; doi:10.1039/d2sc01658c)
Supplement: SC-013-D2SC01658C-s001 [file SC-013-D2SC01658C-s001.pdf]

Electronic Supplementary Material (ESI) for Chemical Science.
This journal is © The Royal Society of Chemistry 2022

Supporting Information

Enhanced C-H Bond Activation by Tuning the Local Environment of Surface Lattice Oxygen of MoO_3

Chenggong Jiang[1], Xin Chang[1], Xianhui Wang[1], Zhi-Jian Zhao[1,2*], and Jinlong Gong[1,2,3,4*]

[1]*Key Laboratory for Green Chemical Technology of Ministry of Education, School of Chemical Engineering and Technology, Tianjin University*

[2]*Collaborative Innovation Center of Chemical Science and Engineering, Tianjin 300072, China*

[3]*Joint School of National University of Singapore and Tianjin University, International Campus of Tianjin University, Binhai New City, Fuzhou 350207, China*

[4]*Haihe Laboratory of Sustainable Chemical Transformations, Tianjin 300192, China*

*Email: zjzhao@tju.edu.cn, jlgong@tju.edu.cn

Contents

S1 Computational details 3

DFT calculation model, method, and U_{eff} value test 5

Crossing potential model details 10

Differential charge density analysis 11

Optimized POM structure and surface H adsorption energy 12

Thermogravimetric analysis 13

Ab initio molecular dynamics simulations 14

Transition state structure details 17

S2 Experimental details 19

Catalyst preparation 19

Characterization 19

Redox performance tests 19

S1 Computational details

Energy, entropy and kinetic information details

The adsorption energy is calculated as follows:

$$\Delta E_{\text{ads}} = E_{\text{adsorbate+ surface}} - E_{\text{surface}} - E_{\text{gas}}$$

The activation energy is calculated as follows:

$$E_a = E_{\text{TS}} - E_{\text{IS}}$$

Where E_a is the activation energy, E_{TS} is the transition state energy and E_{IS} is the initial state energy.

The Gibbs free energy (G) is calculated as follows:

$$G = H - TS = E_{DFT} + ZPE + \int_0^T C_v dT - TS$$

Where H is enthalpy, S is entropy, T is temperature, E_{DFT} is the electron energy calculated by DFT, ZPE is zero-point energy and C_V is isovolumetric heat capacity. C_v and Gas-phase entropies ($S_0^{\text{gas}}(T)$) are taken from the NIST Chemistry Webbook[40]. The adsorption mode of propane was not taken into consideration in free energy analysis.

It is worth noting that some studies have shown that in some cases (at high temperature in particular), the entropy of the adsorbed species may be quite different from that calculated from the vibrational frequency under the harmonic approximation. Campbell et al. reported that under low coverage, the entropy of molecular adsorbate on the plane can be expressed as semiempirical Campbell-Sellers equation [41] [42] (The entropies of all adsorbed state species are calculated using the following equation):

$$S_0^{\text{ad}}(T) = 0.70 S_0^{\text{gas}}(T) - 3.3R$$

The rate constant (k) of the rate determining step (RDS) is calculated by Eyring–Polanyi equation [43] as follows:

$$k = \frac{\kappa k_B T}{h} e^{-\frac{\Delta G^{\dagger}}{RT}}$$

where $\Delta G^{\ddagger}$ is the Gibbs free energy of activation, κ is the transmission coefficient (often assumed to be equal to one), k_B is Boltzmann's constant, and h is Planck's constant.

DFT calculation model, method, and U_{eff} value test

To determine the appropriate van der Waals correction, some common van der Waals correction functionals (Grimme D2 [27], Grimme D3 [28], vdW-DF2 [29], BEEF-vdW [30]) were tested by using the hybrid functional HSE06[31, 32] and the lattice constants measured experimentally [33] as the benchmark. The bilayers of α-MoO_3 (010) crystal face are connected by van der Waals force in z-axis direction, so the difference between different van der Waals correction functionals is mainly reflected in z-axis (i.e., b direction). As a result, the Grimme D2 correction was identified as the most appropriate van der Waals correction. (Figure S1 and Table S1)

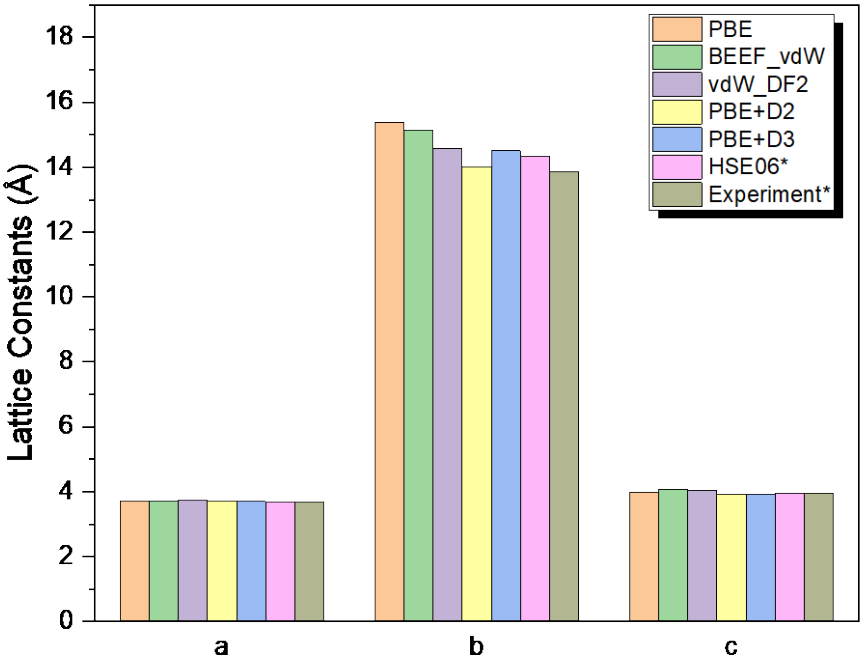

Fig. S1 Optimized lattice constants from different functionals calculation and experimental value.

Table S1 Optimized lattice constants from different functionals calculation and experimental value.

Method	Lattice Constants (Å)		
	a	b	c
PBE	3.72	15.38	3.97
BEEF_vdW	3.71	15.13	4.06
vdW_DF2	3.75	14.58	4.05
PBE+D2	3.71	14.01	3.93
PBE+D3	3.71	14.50	3.92
HSE06*	3.69	14.33	3.95
Experiment*	3.69	13.86	3.96

*: the benchmark value of calculation or experiment

Table S2 The detailed structure information of α-MoO_3 (010)

Bond	Bond length/ Å	E_{vac}/ eV	E_H/ eV
Mo=Ot	1.69	1.92	-0.19
Mo-Oa-Mo	1.78 (left)<br>2.17 (right)	2.04	-0.48
Mo-Os-Mo	1.96	2.86	-0.06

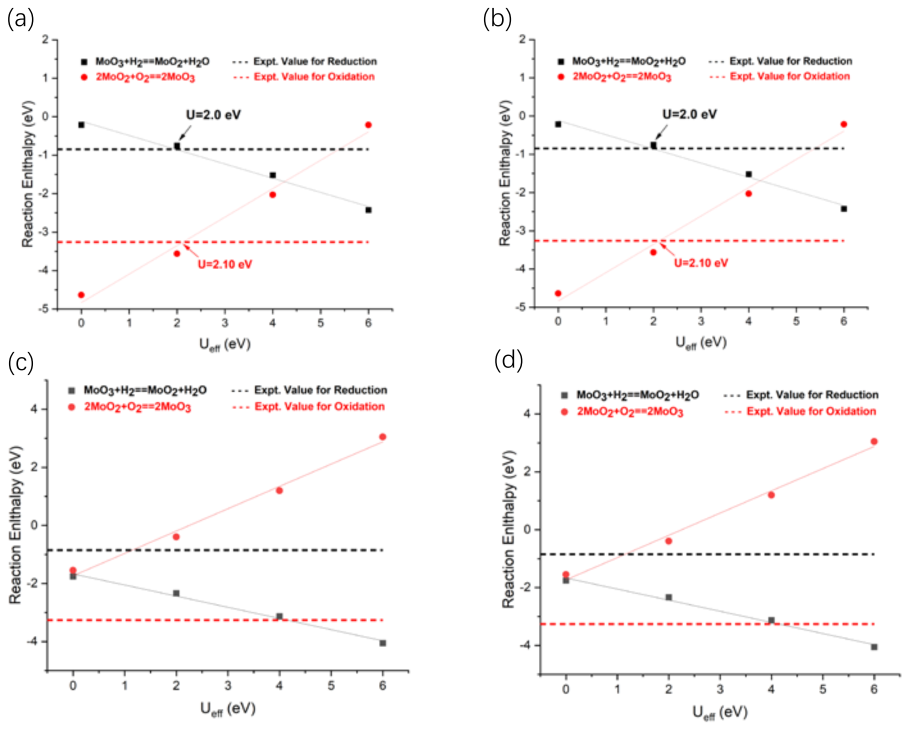

Fig. S2 Fitting the enthalpy of formation and reduction of MoO_3 with different U_{eff} values. (a)PBE; (b) PBE+D2; (c) vdW-DF2; (d) BEEF-vdW

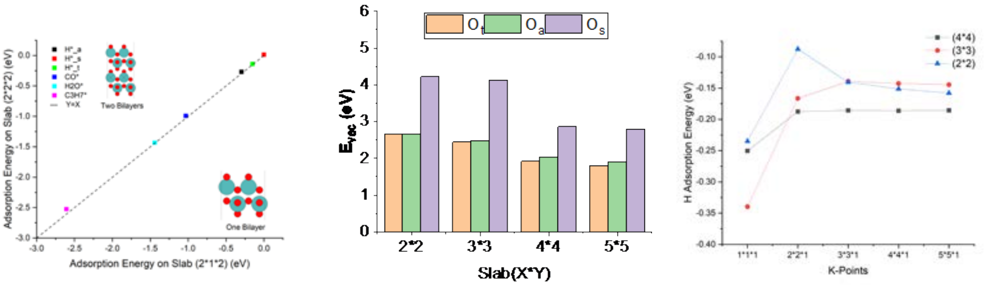

Fig. S3. Determining the appropriate model size: (a) Z-axis; (b) XY-plane and (c) k-point convergence test.

Crossing potential model details

The crossing potential model is an approximate calculation based on the accurate transition state structure. Between the optimized stable initial state structure (C_3H_8*) and the transition state structure(C_3H_7···H), a series of linear interpolation points are obtained by NEB method to simulate the dynamic process of propane C-H bond activation and adsorbate induced surface reconstruction from the initial state to the transition state. The single point energy calculation of these structures is carried out to draw the physisorption potential function plot.

Similarly, the same single point energy calculation was performed between the transition state structure (C_3H_7···H) and the optimized radical structure (C_3H_7 (g) + H*) to plot the chemisorption potential function curve.

Differential charge density analysis

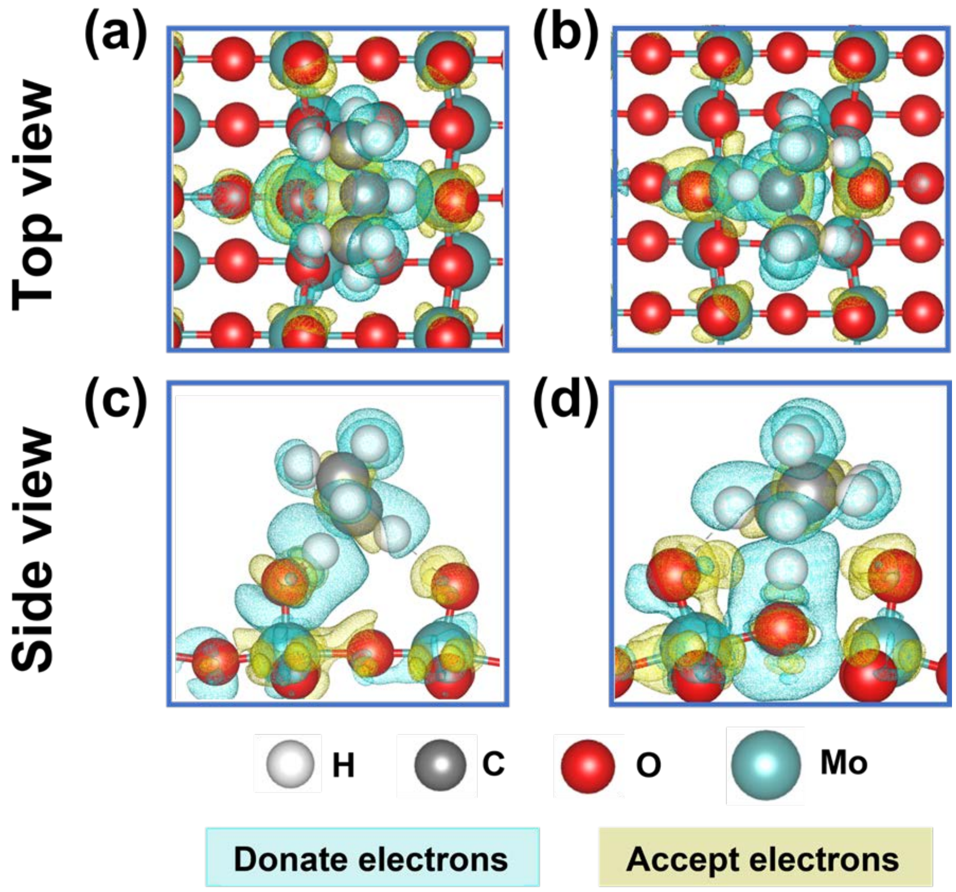

Fig S4. Differential charge density plot for C-H bond activation of propane methylene on α-MoO_3. Mo-O_a-Mo: (a) top view; (c) side view. Mo=O_t: (b) top view; (d) side view.

In order to show more clearly the electron transfer processes occurring on the metal-oxygen species during the C-H bond activation, we have added a differential charge analysis (**Fig. S5**) of the propane C-H bond activation transition state on two lattice oxygens (Mo=O_t and Mo-O_a-Mo) on MoO_3. We can see that during C-H bond activation, H first transfers electrons to the lattice oxygen, which transfers electrons to the metal cation.

Optimized POM structure and surface H adsorption energy

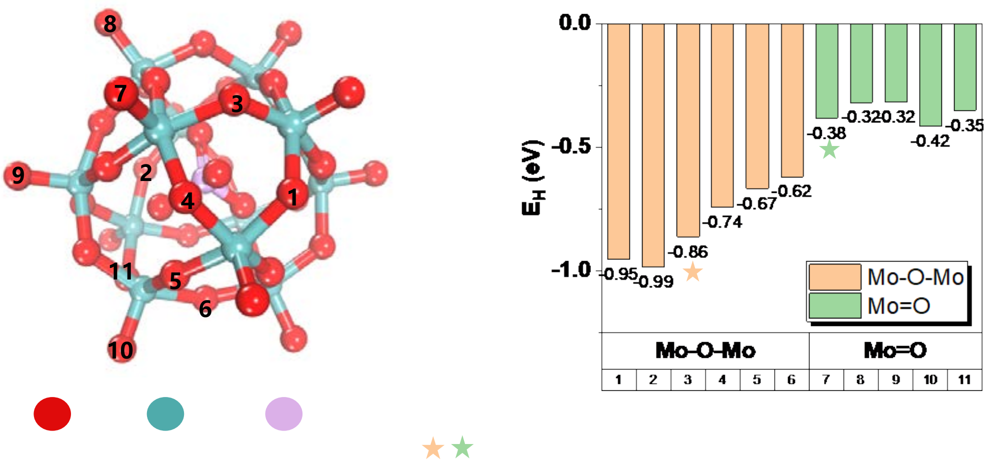

Fig. S5 (a) Optimized structure of POM; (b) the adsorption energy of H on different active sites.

Thermogravimetric analysis

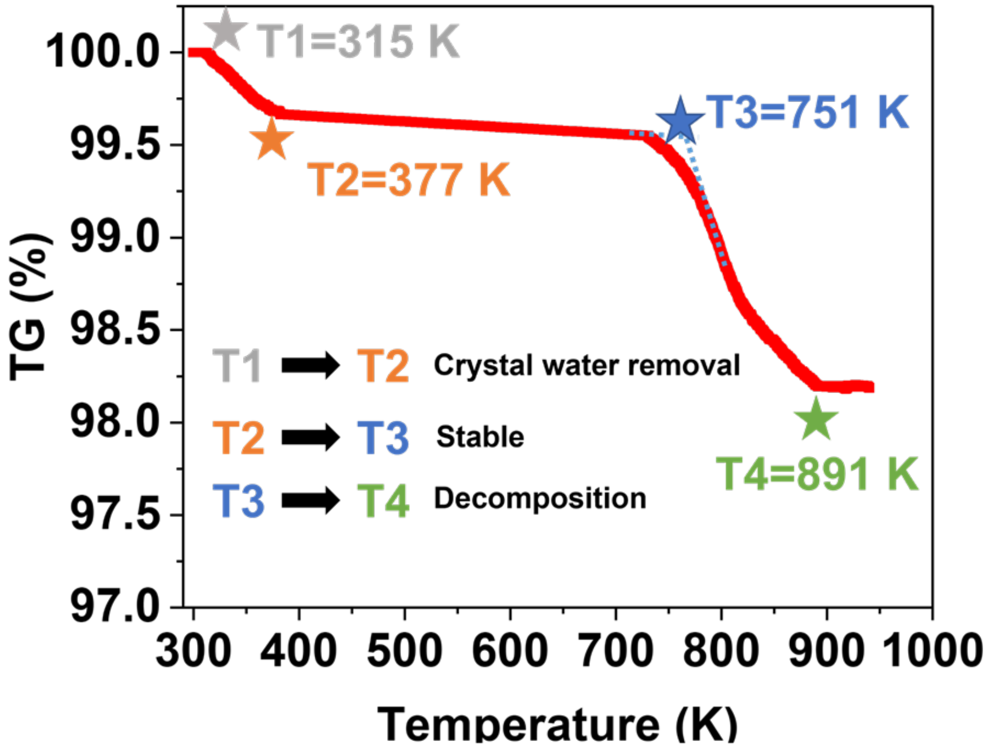

Fig. S6 Thermogravimetric analysis for 20 wt% $H_3PMo_{12}O_{40}/Al_2O_3$. (from 300 K to 950 K)

Ab initio molecular dynamics simulations

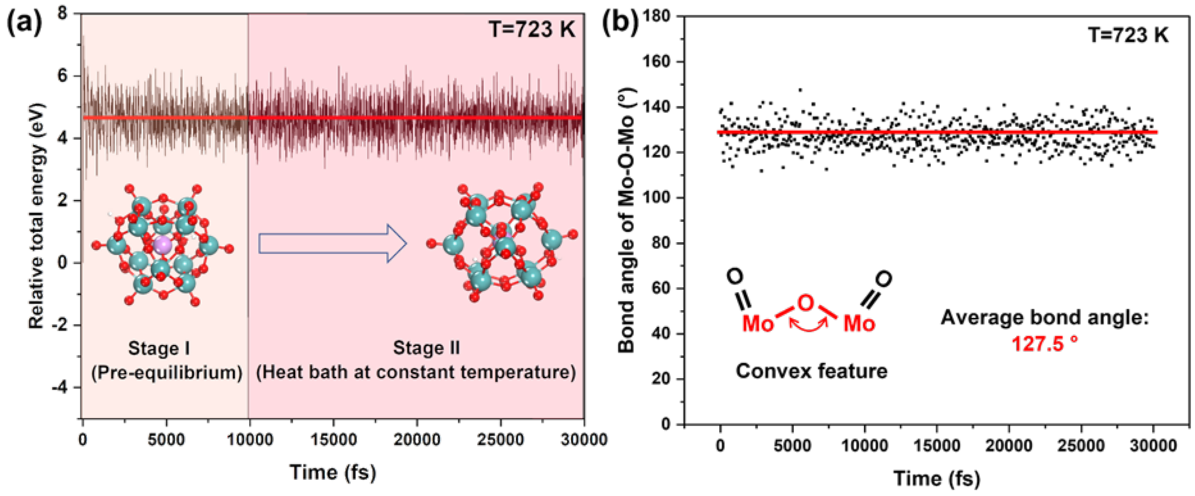

Fig. S7 Ab initio molecular dynamics simulations (AIMD) of $H_3PMo_{12}O_{40}$ at 723 K and NVT ensemble. (a) Total energy dynamic changes; (b) Dynamic monitoring of Mo-O-Mo bond angles.

Table S3. Comparable structure of α-MoO_3 and POM ($H_3PMo_{12}O_{40}$)

Information	α-MoO_3	POM
Coordination number (Mo)	6	6
	Octahedron	Octahedron
Bader charge (Mo)	+2.61 (±0.06)	+2.59 (±0.11)
Surface lattice oxygen	Mo=O; Mo-O-Mo	Mo=O; Mo-O-Mo
Surface topography	Crystal particle	Cluster
Local environment (Mo-O-Mo)	Concave	Convex

Table S4. Calculation details for Gibbs's free energy (ΔG) (T=723 K)

Species	Catalyst Active Site	$E_{\text{-DFT}}$ (eV)	ZPE (eV)	ΔH (eV)	T×ΔS (eV)	ΔG (eV)
C_3H_8(g)	——	0.00	2.74	3.37	2.85	0.51
C_3H_7…H (TS)	α-MoO_3 Mo=O	1.16	2.55	4.30	1.71	2.59
C_3H_7…H (TS)	$H_3PMo_{12}O_{40}$ Mo-O-Mo	1.06	2.54	4.22	1.71	2.51

Transition state structure details

Table S5 All transition state structure details

Reactant	Catalyst	Active site	d(C-H)/Å	Structure	C-H Activation Energy/ eV
CH_4	α-MoO_3 (010)	Mo=O_t	1.49	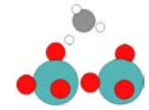	1.87
CH_4	α-MoO_3 (010)	Mo-O_a-Mo	1.38	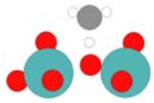	1.98
CH_4	α-MoO_3 (010)	Mo-O_s-Mo	1.49	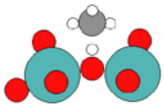	2.28
C_2H_6	α-MoO_3 (010)	Mo=O_t	1.41	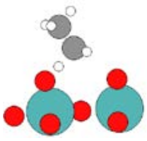	1.41
C_2H_6	α-MoO_3 (010)	Mo-O_a-Mo	1.33	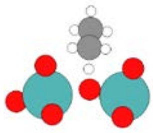	1.58
C_2H_6	α-MoO_3 (010)	Mo-O_s-Mo	1.44	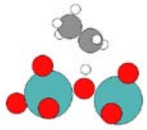	1.91
C_3H_8	α-MoO_3 (010)	Mo=O_t	1.39	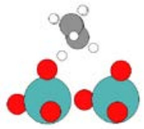	1.15
C_3H_8	α-MoO_3 (010)	Mo-O_a-Mo	1.33	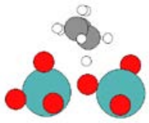	1.39
C_3H_8	α-MoO_3 (010)	Mo-O_s-Mo	1.37	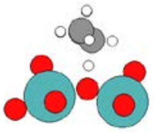	1.74
CH_4	POM	Mo=O	1.51	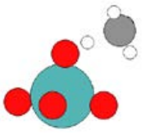	1.91

CH_4	POM	Mo-O-Mo	1.40	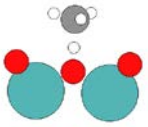	1.59
C_2H_6	POM	Mo=O	1.43	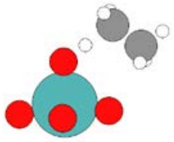	1.58
C_2H_6	POM	Mo-O-Mo	1.34	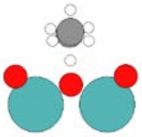	1.27
C_3H_8	POM	Mo=O	1.39	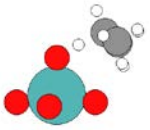	1.28
C_3H_8	POM	Mo-O-Mo	1.31	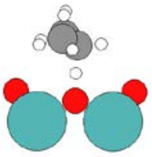	1.06
C_3H_8	α-MoO_3 (010)	Mo=O_t	1.51	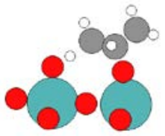	1.22
C_3H_8	α-MoO_3 (010)	Mo-O_a-Mo	1.46	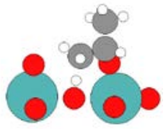	1.14

S2 Experimental details

Catalyst preparation

MoO_3 (99.5%, Meryer Technologies Co., Ltd.) was employed as the reference catalyst without further treatment. The Al_2O_3 supported $H_3PMo_{12}O_{40}$ catalysts with different $H_3PMo_{12}O_{40}$ loading (20 %) were prepared by a simple wet impregnation method. Typically, desired amount of $H_3PMo_{12}O_{40}$ (AR, Tianjin Guangfu Technology Development Co., Ltd.) was dissolved into 5 ml deionized water. Then the solution was drop wise to 1 g of γ-Al_2O_3 powder (Adamas, 99.99%, SBET = 180 m^2/g). The mixture was shook and kept with continuous ultrasonic concussion for 1 h. The supported POM catalysts were obtained after drying in air overnight.

Characterization

X-ray powder diffraction (XRD) patterns were recorded using Rigaku C/max-2500 diffractometer with graphite filtered Cu Kα radiation (λ = 1.5406 Å) in the 2θ range from 10 to 50°. Transmission electron microscopy (TEM) was operated on a JEM-F200 transmission electron microscope at a working voltage of 100 kV. Liquid nitrogen cooled energy-dispersive X-ray spectroscopy (EDS) detector was employed for elemental analysis

Thermal gravity analyses were operated on Setaram Themys thermogravimetry apparatus. Typically, 20 mg of the 20 wt% POM/Al_2O_3 redox catalysts were pre-heated in the suspend crucible at 473 K for 30 minutes under N_2 flow to evaporate the crystallized water. After cooled to room temperature, the sample was then heated to 950 K with a rate of 10 K/min under N_2 flow and the TG profile was recorded with temperature increasing. To eliminate the buoyancy and drag effects, the weight loss experiment was conducted with a blank experiment using the blank crucible at same reaction conditions.

Redox performance tests

The dehydrogenation activity reaction was operated in a quartz fixed-bed reactor 8 mm in ID and 24 cm in length. 0.5 g catalysts (20-40 mesh) diluted with 1 g of quartz particles were loaded in the quartz reactor under atmospheric pressure. The catalysts were heated to the required temperature (723 K) under nitrogen flow (17 mL/min). Afterward, propane flow (4 mL/min) was introduced. The Gas Hour Space Velocity (GHSV) was around 2500 h^{-1}. The outlet gas was analyzed by on-line gas chromatography (GC 2060) equipped with a flame ionization detector (Chromosorb 102 column) and a thermal conductivity detector (Al_2O_3 Plot column). The

instantaneous propane conversion and propylene selectivity at 5th minute was calculated based on equation (1) and equation (2)

$$\text{Con (\%)} = 100 \times ([F_{C3H8}]_{inlet} - [F_{C3H8}]_{outlet}) / [F_{C3H8}]_{inlet}. \quad (1)$$

$$\text{Sel (\%)} = 100 \times [F_{C3H6}]_{outlet} / ([F_{C3H8}]_{inlet} - [F_{C3H8}]_{outlet}) \quad (2)$$

F_{C3H8} and F_{C3H6} are ascribed to the mole flow rate of propane and propylene, respectively.
